# Response of the Rumen Microbiota of Sika Deer (*Cervus nippon*) Fed Different Concentrations of Tannin Rich Plants

**DOI:** 10.1371/journal.pone.0123481

**Published:** 2015-05-08

**Authors:** Zhipeng Li, André-Denis G. Wright, Hanlu Liu, Zhongyuan Fan, Fuhe Yang, Zhigang Zhang, Guangyu Li

**Affiliations:** 1 Jilin Provincial Key Laboratory for Molecular Biology of Special Economic Animals, Institute of Special Animal and Plant Sciences, Chinese Academy of Agricultural Sciences, Changchun, Jilin, China; 2 State Key Laboratory of Genetic Resources and Evolution, Kunming Institute of Zoology, Chinese Academy of Sciences, Kunming, Yunnan, China; 3 School of Animal and Comparative Biomedical Sciences, University of Arizona, Tucson, Arizona, United States of America; University of Wisconsin-Madison, UNITED STATES

## Abstract

High throughput sequencing was used to examine the rumen microbiota of sika deer fed high (OLH) and low concentration (OLL) of tannin rich oak leaves. The results showed that *Prevotella* spp. were the most dominant bacteria. The most predominant methanogens were the members of the order Methanoplasmatales. The dominant rumen protozoa were *Entodinium longinucleatum*, *Eudiplodinium maggii*, and *Epidinium caudatum*, and the fungal communities were mostly represented by *Piromyces* spp. Moreover, the relative abundance of *Pseudobutyrivibrio* spp. (P=0.026), unidentified bacteria (P=0.028), and *Prevotella* spp. (P=0.022) was lower in the OLH group than in the OLL group. The concentration of propionate in the OLH group was greater than in the OLL group (P=0.006). Patterns of relationships showed that methanogens belonging to the order Methanoplasmatales were negatively correlated with *Treponema* spp., *Ent*. *Longinucleatum*, and acetate. *Methanosphaera stadtmanae* was positively correlated to propionate, while *Methanobrevibacter ruminantium* was negatively associated with *Methanobrevibacter thaueri* and *Methanobrevibacter millerae*. Tannins altered the rumen microbes and fermentation patterns. However, the response of the entire rumen microbiota and the relationship between rumen microorganisms and the fermentation parameters were not fully understood.

## Introduction

Plant tannins (condensed and hydrolysable tannins) are ubiquitously distributed in forage trees, shrubs, legumes, and tree bark [1]. Previous studies reported that plant tannins had beneficial effects on protein metabolism, on fermentation patterns such as volatile fatty acids (VFAs), prevention of frothy bloat, and the potential to decrease methane emissions from ruminants [2–4].

The rumen is inhabited by a diverse consortium of microorganisms, including bacteria, archaea, protozoa, fungi, and viruses. Collectively referred to as the rumen microbiota, these microorganisms have a symbiotic relationship with the ruminant, and play a critical role in biomass degradation [5]. Therefore, the altered rumen metabolism is directly linked to the rumen microbiota [6,7]. However, there have been limited studies linking high-throughput sequence data of the rumen microbiota with fermentation patterns in response to feeding diets containing different concentrations of tannins.

A few studies have provided evidence for the relationship between the particular bacterial groups and fermentation products in the rumen. For example, Carberry et al. [8] found negative associations between *Prevotella* spp. and isobutyrate, and between *Prevotella* spp. and isovalerate concentrations in the rumen liquid based on the Spearman's partial correlation. Kittelmann et al. [9] found that there was a positive correlation between the occurrence of the *Methanobrevibacter ruminantium* clade and bacteria in the family Fibrobacteraceae based on the Spearman’s rank correlations. Moreover, occurrence of the *Methanobrevibacter gottschalkii* clade was positively correlated with bacteria in the family Ruminococcaceae. In our previous study, the relationship, or interplay patterns between the bacterial community and the fermentation parameters in the rumen of sika deer fed tannin rich plants: oak leaves (*Xylosma racemosum*; tannin content, 100 mg/1 kg dried matter) based diets, were distinctly different than in the rumen of sika deer fed corn stover or corn silage based diets [10]. However, when the tannin rich plants were fed to sika deer, the interplay between the rumen microbiota, particularly the methanogens, and the fermentation patterns remained poorly understood. Therefore, using high throughput sequencing, we (i) investigated the rumen microbiota of sika deer fed two concentrations of oak leaves rich of tannins, (ii) compared rumen fermentations patterns under these two diets, and (iii) examined the interplay patterns between the rumen microbiota and metabolic phenotypes.

## Materials and Methods

### Animals and sampling

Four rumen-cannulated adult male sika deer (*Cervus nippon*), were used in the present study, and maintained at the research farm (44.04°N, 129.09°E) of the Institute of Special Animal and Plant Sciences, of the Chinese Academy of Agricultural Sciences, in Jilin Province. All animal were housed in individual pens, and all animal procedures were approved and authorized by the Chinese Academy of Agricultural Sciences Animal Care and Use Committee, and by the Institute of Special Animal and Plant Sciences Wild Animal and Plant Subcommittee.

Four sika deer received the different diets in a cross over study that were composed of the same concentrate (64.5% corn, 19.7% soybean meal, 12.8% corn distiller dried grains, and a 3% mixture of vitamins and mineral salts). The concentrate diet was mixed with either of the two different concentrations of oak leaves to form two different diets. The two different ratios of concentrate to oak leaves were 40:60 (OLH, tannin content: 60 mg/1 kg dried matter) and 60:40 (OLL, tannin content: 40 mg/1 kg dried matter). All sika deer were fed twice each day at 0800h and 1600h and had free access to water. After one week of adaption to the diets, sika deer received each diet for 28 days, and the rumen contents were obtained via rumen cannula immediately before the morning feeding at day 29 for 1 day. Rumen samples were stored at -80°C for later analysis.

### DNA extraction

Total genomic DNA of microorganism was extracted from the whole ruminal contents containing solid and liquid fractions of each animal using the QIAamp DNA Stool Mini Kit (QIAGEN, Valencia, CA) according to the manufacturer’s instructions.

### Amplification of target genes and high-throughput sequencing

The bacterial 16S rRNA gene was amplified using primers 27F [11] and 519R [12], the methanogenic 16S rRNA gene was amplified using primers Met86F [13] and 519R [12], the partial 18S rRNA gene of protozoa was amplified using primers GIC1080F and GIC1578R [14], and the internal transcribed spacer region of fungi was amplified using the primers 1737F and 2043R [15]. Each specific primer pair contained the appropriate Illumina adapter sequence, and a 8 bp barcode. The resulting amplicons were purified using QIAquick PCR Purification Kit (QIAGEN, Valencia, CA). The purified amplicons were quantified using QuantiFluor-P Fluorometer (Promega, CA), pooled in equimolar concentrations, and sequenced on Illumina PE MiSeq 300 platform generating paired 300-basepair reads.

### Bioinformatics analysis

The read pairs were extracted and concatenated according to the barcodes for each paired read from each sample generating contigs. Contigs with an average quality <20 over a 10 bp sliding window were culled. The retained contigs were processed and analyzed using QIIME ver. 1.7.0 [16].

Contigs were examined for quality control using the following criteria: the minimum sequence length was 400 nt; the maximum sequence length was 500 nt; minimum quality score for a single nucleotide was 25; the maximum number of errors in the barcode was 0; the maximum length of homopolymer run was 6 (default parameter, this does not affect the interpretations of the data); the number of mismatches in the primer was 0; ambiguous and unassigned characters were excluded. The remaining sequences were clustered into operational taxonomic units (OTUs) using Usearch61 according to the sequence identity of 97% at species level, for the 16S rRNA (bacteria and methanogens), and the ITS region (fungi) [9,17], and the sequence identity of 95% at species level for the 18S rRNA (protozoa) genes. Representative sequences of OTUs were aligned to the Greengenes database for bacteria and methanogen 16S rRNA genes [18], to the Silva database for protozoal 18S rRNA genes [19], and to the Unite ITS database for fungi [20]. Potential chimera sequences were removed using Chimera Slayer [21]. The remaining representative OTUs were screened using Basic Local Alignment Search Tool [22].

OTUs that were found in at least 4 samples (animals) for each community (bacteria, methanogens, protozoa and fungi) were retained for the further analysis. Alpha-diversity from all samples including Shannon-Wiener and Simpson indices were also calculated from QIIME [16]. The sequences were deposited in the Sequence Read Archive under accession number SRP050105.

### Measurement of metabolic phenotypes in the rumen of sika deer fed two diets

Rumen fluid was centrifuged at 15,000 g for 10min at 4°C, and 0.2 ml of 2-ethylbutyric acid (internal standard, 2 g/L) in meta phosphoric acid (25% w/v) was added to 1 ml of clarified rumen fluid. The concentrations of VFAs in the rumen were determined by gas chromatography with a fiame ionization detector and a DB-FFAP column (30 m×0.25 μm ×0.25 μm, Agilent Technologies 6890GC, USA). The carrier gas was N2 at a flow rate of 2.2 ml/min. The analysis was a gradient oven temperature of 80–170°C with an incremental rate of 10°C/min for optimal separation and a detector temperature of 250°C.

### Relationships between rumen microbiota and fermentation parameters

The interactive analysis among bacteria, methanogens, protozoa, fungi and metabolic phenotypes was performed in R package (V3.1.0, http://www.r-project.org/). Spearman’s rank correlations and *P*-values were calculated and plotted using the packages hmisc (http://cran.r-project.org/web/packages/Hmisc), and corrplot (http://cran.r-project.org/web/packages/corrplot). Moreover, the interrelations were constructed using the method as described by Zhang et al. [23]. Statistic *P*-values were corrected using the false discovery rate method of the *p*.*adjust* package in R. Correlations have an absolute Pearson’s correlation above 0.7 with an false discovery rate-corrected significance level under 0.05. These correlations were transformed into links, and were then visualized using Cytoscape 2.8.2 [24].

### Statistical analysis

Statistical analysis was performed using the SigmaPlot 12.0 (Systat Software, Inc.) and R software packages. All variations in different groups were checked for normal distribution using the Shapiro-Wilk test (significance value of *P*<0.05). When normally distributed, multiple samples comparisons were performed using one-way analysis of variance (parametric), and using Kruskal-Wallis analysis on ranks (non-parametric) for un-normal distribution with the significant value of *P*<0.05. All parameters were expressed as the mean and standard error of each group.

## Results

### Summary of high-throughput sequencing data

A total of 783,651 high-quality sequences were generated. Based on the 97% sequence identity, 249,393 bacterial sequences were assigned to 1,249 OTUs, 282,503 methanogen sequences were assigned to 76 OTUs, 138,597 rumen ciliate sequences were assigned to 9 OTUs, and 39,349 fungal sequences were assigned to 5 OTUs. The results of Good’s coverage [25] showed that 98%-100% of the microbial species were sampled for the four groups of microorganisms, indicating that the sampling effort had sufficient sequence coverage for each microbial group (Table 1).

**Table 1:** Number of sequences, OTUs, and alpha-diversity indices in OLH and OLL groups.

Taxa	Groups	Seq/ sample	OTUs /sample	Goods Coverage	Shannon	Simpson
Bacteria	OLH	30,113	1,004	0.99	7.44	0.96
	OLL	32,234	999	0.99	7.69	0.98
Methanogen	OLH	31,319	76	0.99	3.51	0.85
	OLL	39,306	76	0.99	3.18	0.79
Protozoa	OLH	19,666	7	1.00	1.68	0.62
	OLL	14,938	6	1.00	1.23	0.42
Fungi	OLH	5,662	5	1.00	0.70	0.27
	OLL	4,176	4	1.00	0.73	0.29

OLH: High group; OLL: Low group

### Microbial communities in the OLH and OLL groups

**Fig 1:**
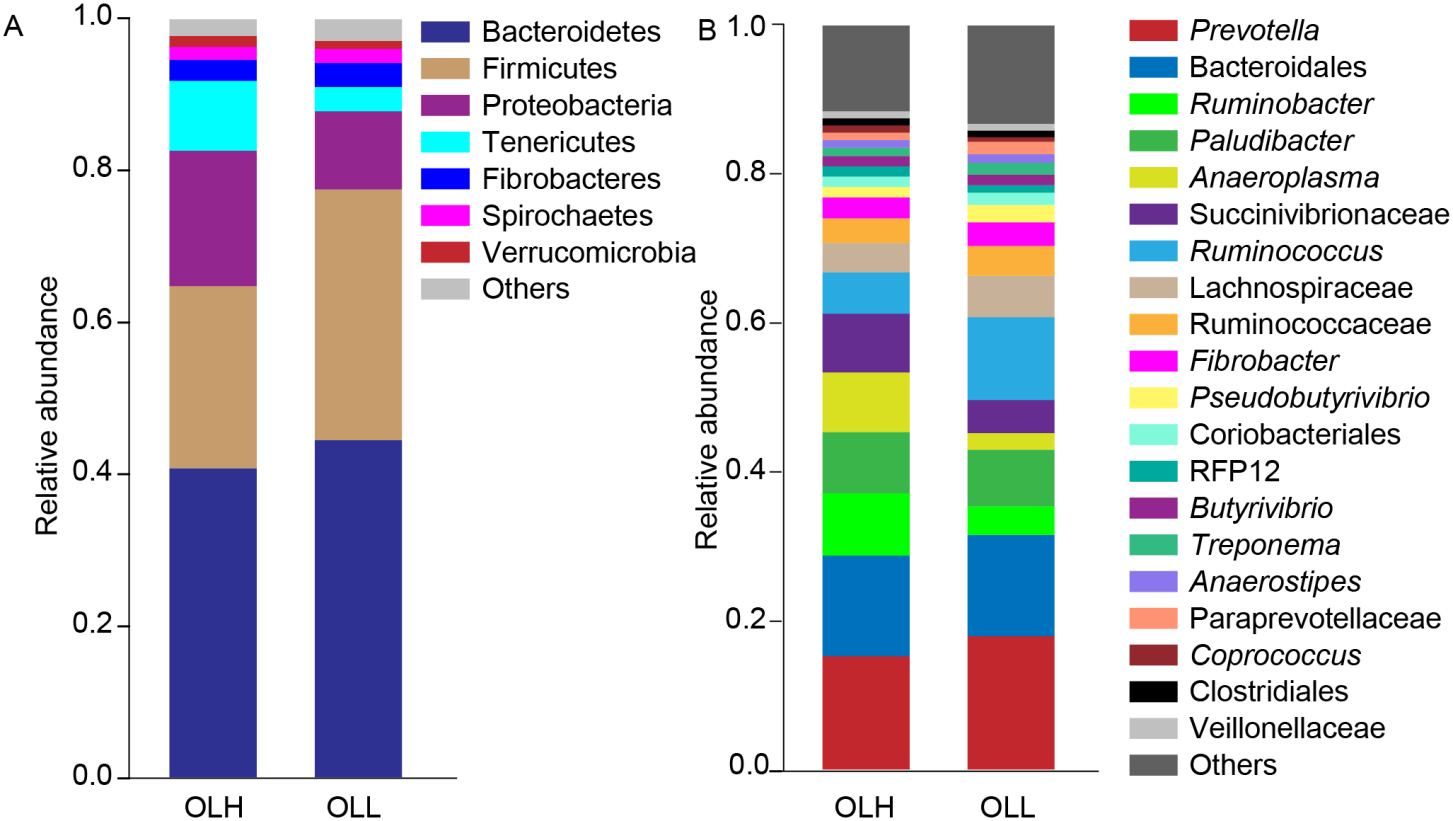
The bacterial community composition in the two groups at phylum level (A) and genus level (B). OLH = High group, OLL = Low group. The relative abundance of Firmicutes*, Pseudobutyrivibrio*, and unidentified bacteria and *Prevotella* belonging to the family Paraprevotellaceae* was significantly different between the two groups, with * means the significance at *P*<0.05.

Overall, 1,249 bacterial OTUs represented 18 phyla and 94 genera. On average, the top three bacterial phyla in the two diet groups were Bacteroidetes (Mean±S.E: 42.7±2.1%), Firmicutes (28.5±5.6%) and Proteobacteria (14.1±2.6%) (Fig 1A). However, the distribution of Firmicutes in the OLH group (24.0±3.1%) was lower than in the OLL group (33.0±1.8%) (*P* = 0.039). Moreover, the relative abundance of bacteria belonging to the phyla Proteobacteria and Tenericutes tended to increase in the OLH group (17.8±4.3% and 9.2±4.3%, respectively) as compared to the OLL group (10.3±2.3% and 3.2±0.9%, respectively).

At the genus level, *Prevotella* spp. was the most dominant bacteria, accounting for 15.3±0.3% and 18.0±1.6% in the OLH and OLL groups, respectively, followed by unidentified bacteria within the order Bacteroidales (OLH:13.5±2.1%; OLL: 13.6±2.0%) and *Paludibacter* spp. (OLH: 8.2±2.0%; OLL: 7.6±1.3%) (Fig 1B). The relative abundance of *Pseudobutyrivibrio* spp. (*P* = 0.026), unidentified bacteria (*P* = 0.028), and *Prevotella* spp. (*P* = 0.022) belonging to the family Paraprevotellaceae were lower in the OLH group (1.4±0.2%, 0.9±0.1% and 0.09±0.04%, respectively) than in the OLL group (2.4±0.2%, 1.7±0.2% and 0.3±0.04%, respectively). Moreover, the proportion of *Ruminobacter* spp. (8.4±2.4%), *Anaeroplasma* spp. (8.0±4.8%) and unidentified bacteria within the family Succinivibrionaceae (7.9±2.3%) tended to increase in the OLH group in comparison to the OLL group (3.8±1.7%, 2.3±0.7% and 4.4±1.3%, respectively), but *Ruminococcus* spp. was decreased (OLH: 5.6±0.9%; OLL: 11.1±2.8%).

**Fig 2:**
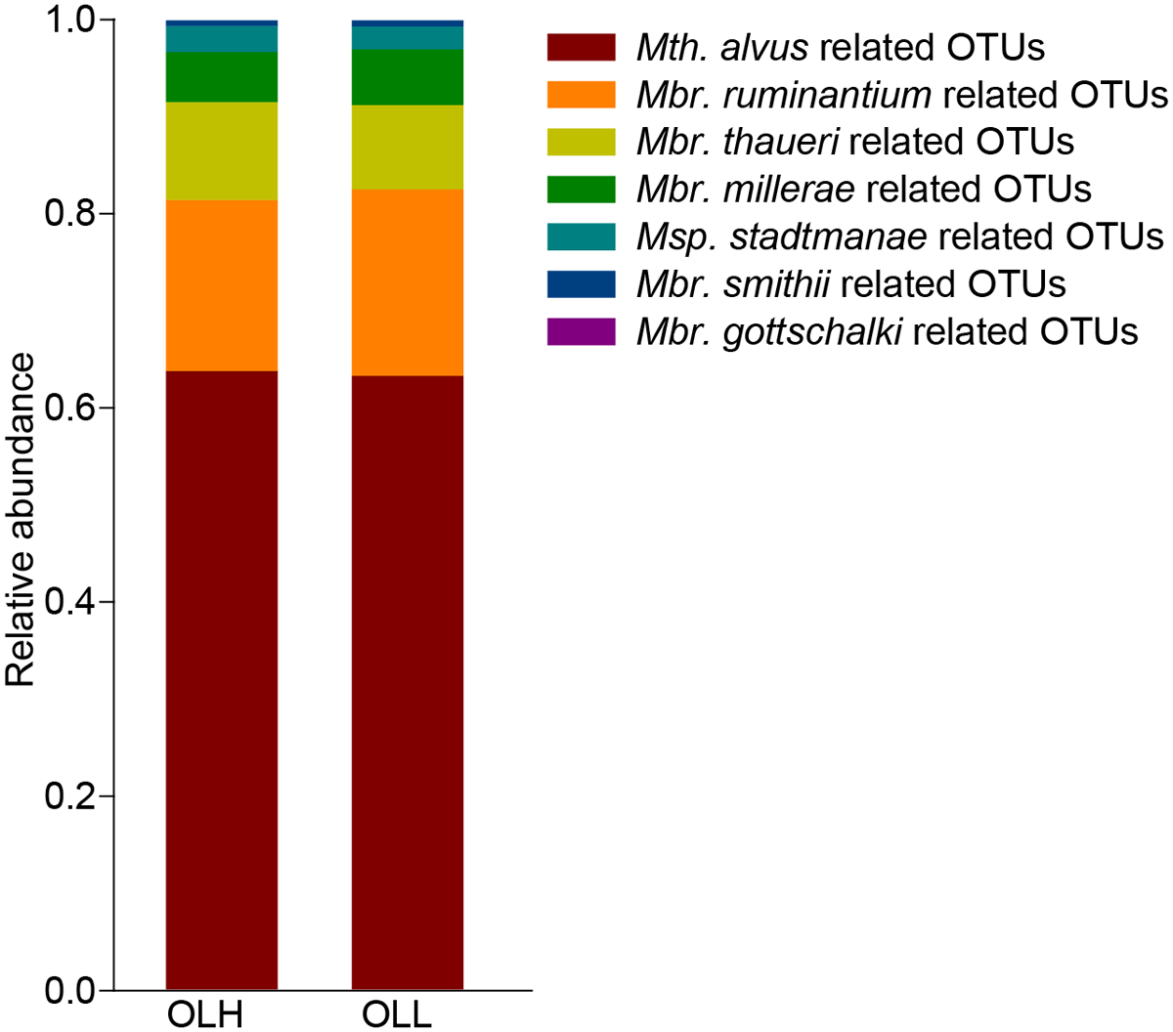
The composition of rumen methanogens in the two groups. *Mbr* = *Methanobrevibacter*, *Msp* = *Methanosphaera*, *Mth* = *Methanomethylophilus*, OTUs = operational taxonomic units, OLH = High group, OLL = Low group.

For the methanogens, all 76 OTUs in the OLH and OLL groups were assigned to two orders: Methanobacteriales (OLH: 36.2±1.9%; OLL: 36.7±2.3%) and Methanoplasmatales (OLH: 63.8±2.2%; OLL: 63.3±2.6%). In order to examine methanogen composition at the species level, these OTU sequences were examined against the NCBI nr database using Basic Local Alignment Search Tool [22]. *Methanobrevibacter* spp. accounted for 33.6±2.0% (OLH) and 34.3±2.7% (OLL), and *Methanosphaera* spp. accounted for 2.6±0.1% (OLH) and 2.4±0.6% (OLL). Moreover, *Methanobrevibacter ruminantium* (OLH: 17.6±5.6%; OLL: 19.2±6.8%) and *Methanobrevibacter thaueri* (OLH: 10.1±6.1%; OLL: 8.7±4.7%) were the most prevalent phylotypes, followed by *Methanobrevibacter millerae* (OLH: 5.1±1.2%; OLL: 5.7±0.6%). All OTUs within the order Methanoplasmatales were similar to Candidatus *Methanomethylophilus alvus* with the 98%-100% sequence identity (Fig 2).

**Fig 3:**
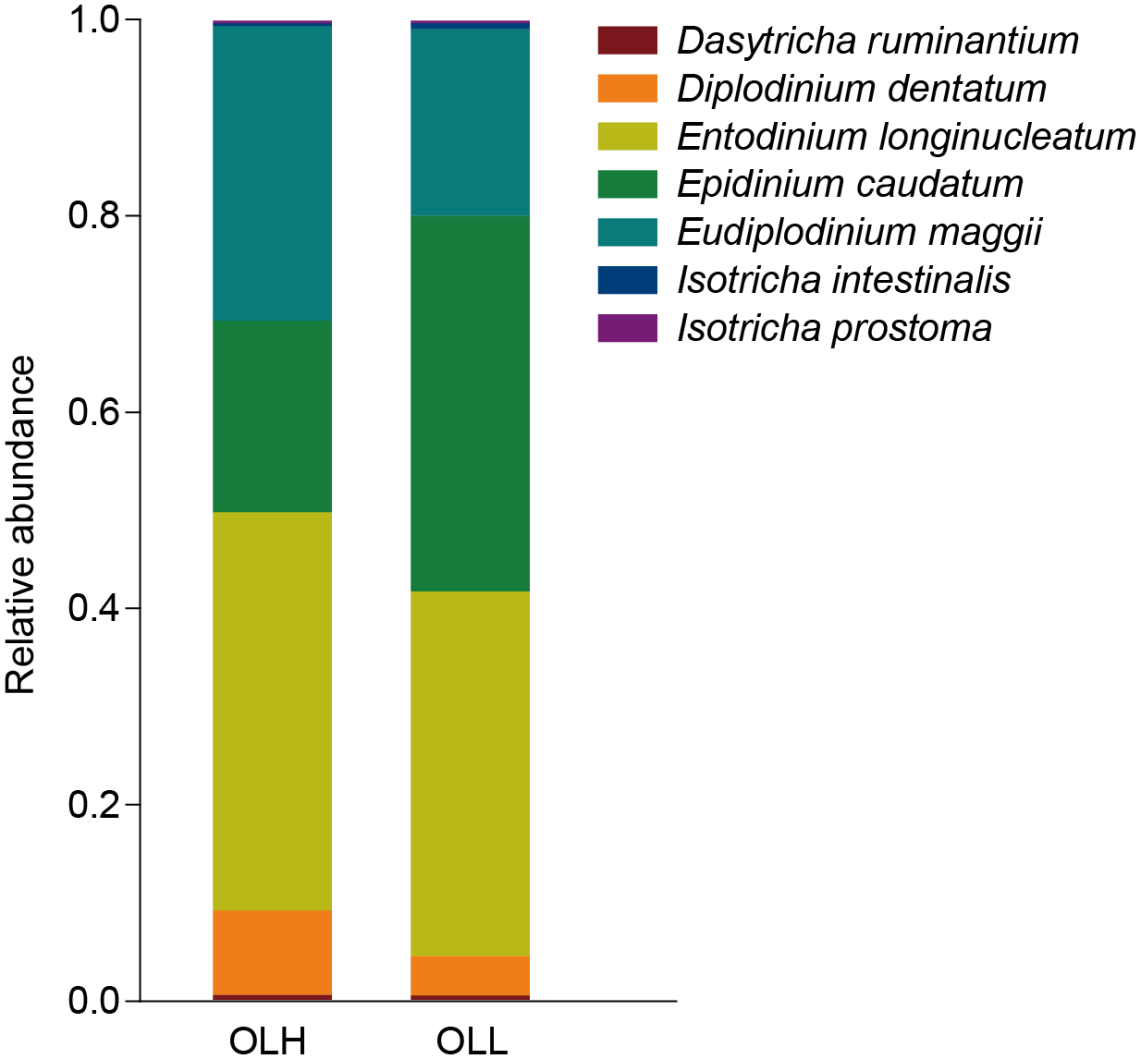
The composition of rumen protozoa in the two groups. OLH = High group, OLL = Low group.

For the protozoa, *Entodinium longinucleatum* accounted for 40.6±7.2% of the rumen protozoa in the OLH group, followed by *Eudiplodinium maggii* (30.0±5.9%), and *Epidinium caudatum* (19.5±8.8%). While, *Ep*. *caudatum* accounted for 38.3±2.6% in the OLL group, followed by *Ent*. *longinucleatum* (37.1±8.5%) and *Eud*. *maggii* (19.0±3.6%) (Fig 3).

**Fig 4:**
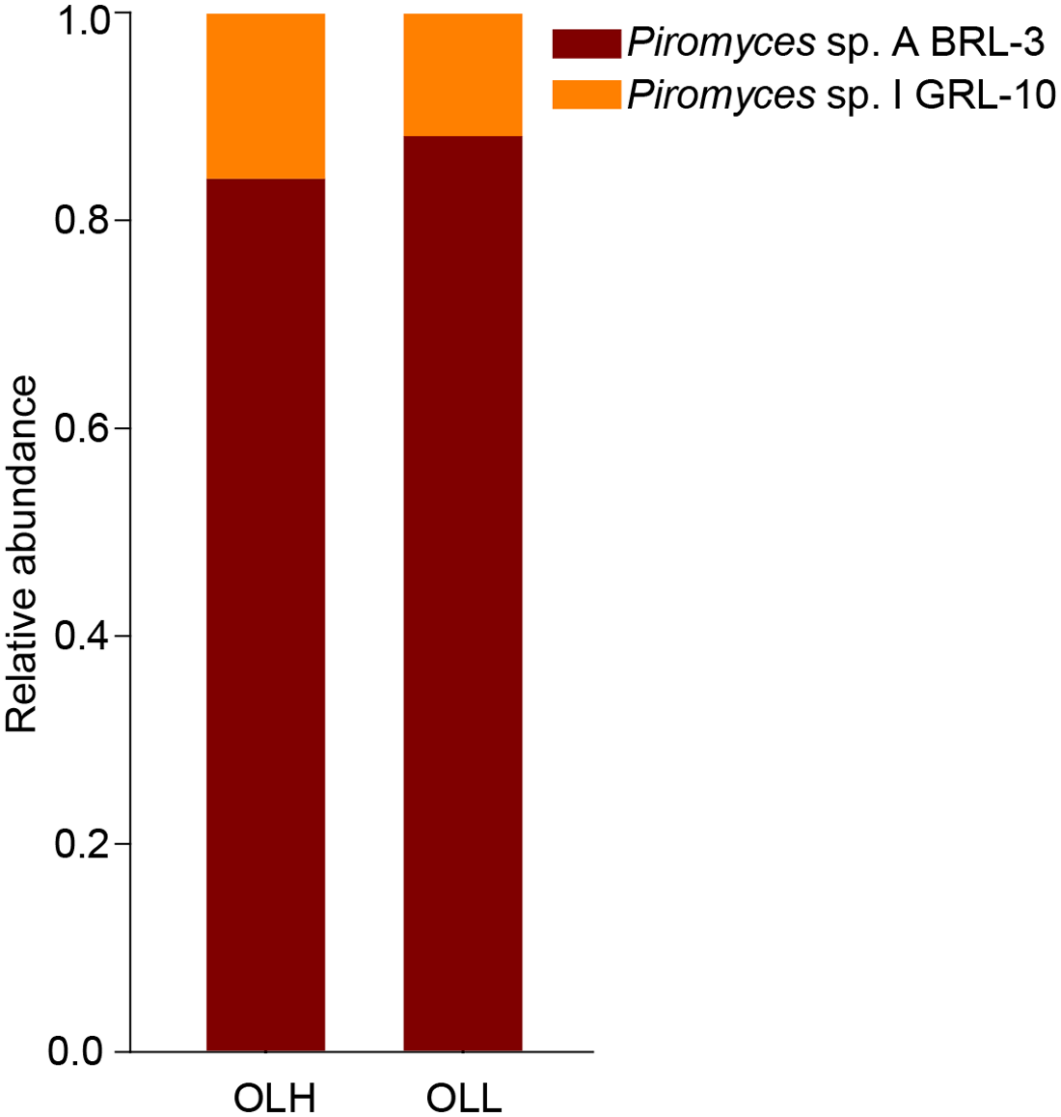
The composition of rumen fungi in the two groups. OLH = High group, OLL = Low group.

For the fungi, *Piromyces* sp. A-BRL-3 was the most abundant in the OLH (84.0±6.3%) and OLL (88.1±4.9%) groups, followed by *Piromyces* sp. I-GRL-10 (OLH: 16.0±4.8%; OLL: 11.9±2.9%) (Fig 4).

### Composition of VFAs in the rumen of sika deer from the OLH and OLL groups

As shown in Table 2, the concentration of propionate, in the OLH group (10.14±0.79 mmol/L) was greater than in the OLL group (7.44±0.47 mmol/L, *P* = 0.006), and the ratio of acetate to propionate in the OLH group (3.91±0.18) was remarkably decreased as compared to the OLL group (4.85±0.16, *P* = 0.003).

**Table 2:** Comparing the concentrations of volatile fatty acids in the OLH and OLL groups.

VFAs(mmol/L)	OLH Mean ± S.E	OLL Mean ± S.E	*P* value
Acetate	39.67±1.77	35.98±2.34	0.778
Propionate	10.14±0.79	7.44±0.47	0.006
Isobutyrate	0.53±0.02	0.47±0.045	0.264
Butyrate	5.16±0.21	4.60±0.45	0.27
Isovalerate	0.69±0.022	0.57±0.078	0.161
Valerate	0.52±0.027	0.49±0.016	0.258
TVFAs	56.70±2.43	49.53±3.20	0.085
Acetate/Propionate	3.91±0.18	4.85±0.16	0.003

VFAs, volatile fatty acids; TVFAs, total volatile fatty acids; S.E: Standard Error; OLH: High group; OLL: Low group

### Relationships between rumen microbiota and metabolic phenotypes

**Fig 5:**
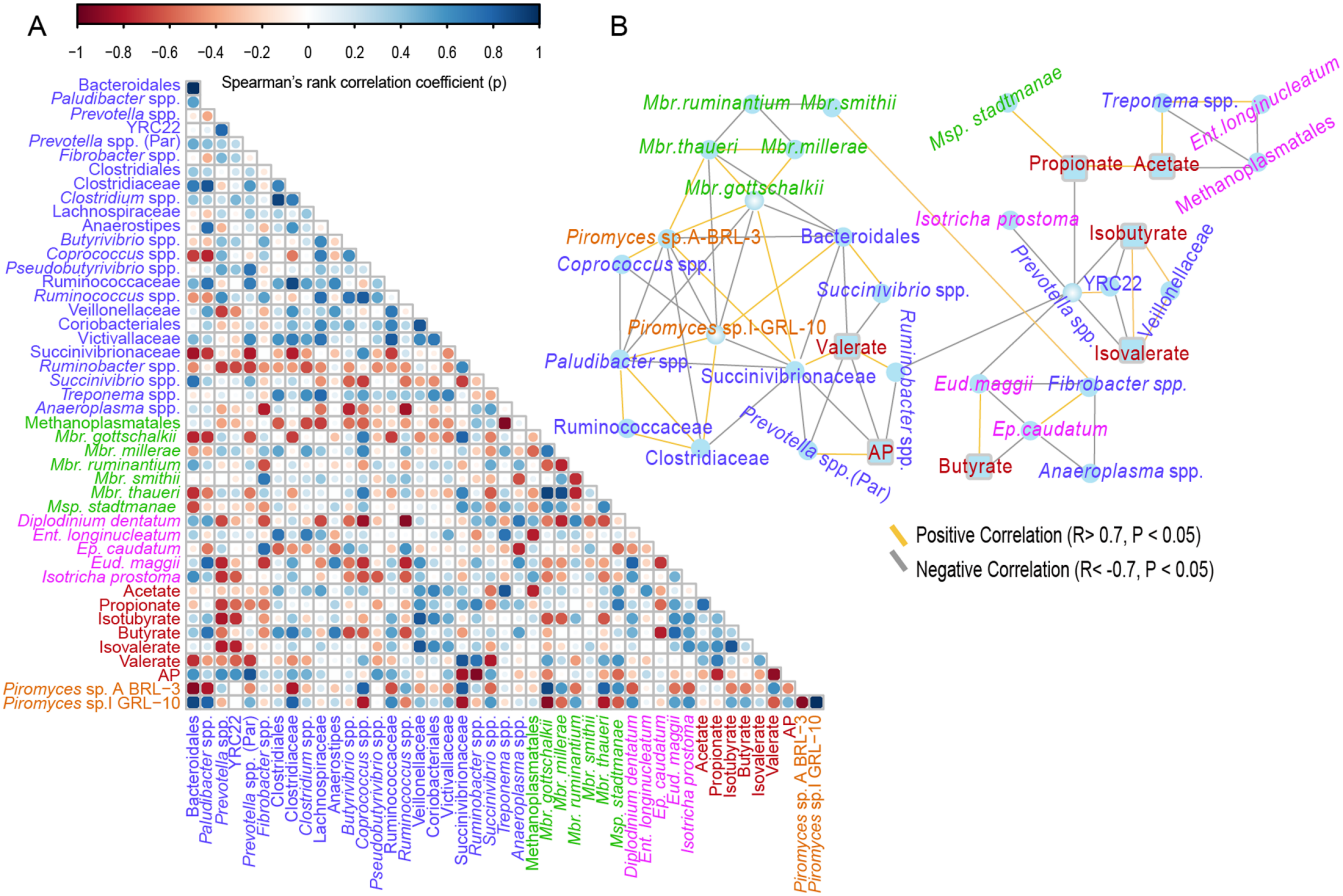
Relationships between microbial populations and fermentation products in the rumen of sika deer fed tannin rich plants. (A) Correlation between microbial populations and fermentation products. Strong correlations are indicated by large circles, whereas weak correlations are indicated by small circles. The colors of the scale bar denote the nature of the correlation with 1 indicating perfect positive correlation (dark blue) and -1 indicating the negative correlation (dark red). (B) Co-occurrence network analysis among microbial populations and fermentation products. Bright blue circle nodes represent microbial populations at genus level, and rounded rectangle nodes represent fermentation products. Each co-occurring pair among microbial populations at genus level and fermentation products has an absolute Spearman rank correlation above 0.70 [Gold line: positive correlation (R >0.70); Gray line: negative correlation (R <-0.70)] with an false discovery rate-corrected significance level under 0.05. Different groups of microorganisms and fermentation products were indicated by various colors: Bacteria (blue), Methanogens (green), Protozoa (purple), Fungi (orange), Fermentation products (red). Par = Paraprevotellaceae, *Mbr* = *Methanobrevibacter*, *Msp* = *Methanosphaera*, *Ep* = *Epidinium*, *Ent* = *Entodinium*, *Eud* = *Eudiplodinium*.

The results (Fig 5A and 5B) showed that *Methanobrevibacter gottschalkii* was positively correlated to unidentified bacteria belonging to the family Succinivibrionaceae (R = 0.88; *P* = 2.7×10^-15^), *Piromyces* sp. A-BRL−3 (R = 0.97; *P* = 2.9×10^-25^), *Mbr*. *thaueri* (R = 0.96; *P* = 1.9×10^-10^) and *Mbr*. *millerae* (R = 0.79; *P* = 1.3×10^-10^). However, *Mbr*. *gottschakii* was negatively associated with *Paludibacter* spp. (R = -0.90; *P* = 5.6×10^-22^) and *Piromyces* sp.I-GRL-10 (R = -0.97; *P* = 3.5×10^-30^). *Methanobrevibacter ruminantium* was negatively associated with *Mbr*. *thaueri* (R = -0.82; *P* = 8.1×10^-12^), *Mbr*. *millerae* (R = -0.82; *P* = 4.0×10^-12^) and *Mbr*. *smithii* (R = -0.58; *P* = 3.3×10^-5^). Moreover, *Fibrobacter* spp. had a positive interaction with *Mbr*. *smithii* (R = 0.69; *P* = 1.2×10^-7^).

*Epidinium caudatum* was negatively (R = -0.79; *P* = 0.02) associated with butyrate concentrations, whereas *Eud*. *maggii* was positively (R = 0.80; *P* = 0.01) associated with butyrate concentrations. Methanogens belonging to the order Methanoplasmatales were negatively correlated to acetate concentration (R = -0.65; *P* = 1.2×10^-6^), *Treponema* spp. (R = -0.93; *P* = 1.6×10^-23^), and *Ent*. *longinucleatum* (R = -0.73; *P* = 1.1×10^-8^), whereas *Methanosphaera stadtmanae* positively interacted with propionate concentrations (R = 0.73; *P* = 1.3×10^-8^). *Prevotella* spp. negatively interacted with *Ruminobacter* spp. (R = -0.72; *P* = 2.4×10^-8^), *Eud*. *maggii* (R = -0.88; *P* = 3.6×10^-15^), propionate concentrations (R = -0.71; *P* = 6.0×10^-8^), isobutyrate (R = -0.85; *P* = 7.7×10^-14^) and isovalerate concentrations (R = -0.81; *P* = 2.1×10^-11^). However, unidentified bacteria within the family Veillonellaceae showed positive relationships with isovalerate (R = 0.91; *P* = 8.3×10^-25^) and isobutyrate (R = 0.89; *P* = 2.2×10^-16^) concentrations, respectively. Valerate concentrations were negatively related to *Succinivibrio* spp. (R = -0.85; *P* = 1.6×10^-13^), *Prevotella* spp. belonging to the family Paraprevotellaceae (R = -0.86; *P* = 4.4×10^-14^) and ratio of acetate to propionate (R = -0.86; *P* = 1.3×10^-13^), but positively related to *Ruminobacter* spp. (R = 0.70; *P* = 1.0×10^-7^), and bacteria belonging to the family Succinivibrionaceae (R = 0.90; *P* = 8.7×10^-29^).

## Discussion

Using high-throughput sequencing, the present study showed the responses of rumen microbiota of sika deer fed tannin rich plants, and revealed the interplay patterns between rumen microbiota and metabolic parameters. These results will help us to understand the beneficial effects of tannins on ruminants and to use tannin rich plants in ruminants farming.

The results revealed that *Prevotella* spp. were the dominant bacteria in the rumen of sika deer fed oak leaves, consistent with the results of other cervids [26–29]. *Prevotella* spp. made up a large part of the genetic and metabolic diversity in rumen microbial communities [30]. Recent studies also showed that species of *Prevotella* provided the ability to adapt to various diets [31]. The current study demonstrated that *Prevotella* spp. played important roles in the fermentation of oak leaves.

The distribution of Firmicutes (e.g. *Ruminococcus* spp.) in the OLH group was decreased compared to that in the OLL group (*P* = 0.039) indicating that some species belonging to the phylum Firmicutes may be sensitive to tannins [32]. However, bacteria belonging to the family Succinivibrionaceae, which could ferment glucose and other carbohydrates and produce succinate and acetate, were increased in the OLH group. Additionally, the ratio of Firmicutes to Bacteroidetes in the OLH group (0.59) tended to decrease as compared to the OLL group (0.74). Bacteroidetes utilize the succinate pathway via methylmalonyl-CoA to generate propionate [33]. Consequently, the concentrations of propionate in the OLH group were increased as compared to the OLL group. These results suggested that the altered bacterial community composition linked with the production of different VFAs.

The rumens of sika deer in both diets were dominated by methanogens belonging to the order Methanoplasmatales, followed by *Methanobrevibacter* spp. Similarly, Min et al. [34] also found that *Methanobrevibacter* spp. was decreased with the supplementation of tannins in feces of goat. Additionally, previous studies showed that methanogens belonging to the order Methanoplasmatales were more abundant in the free-living community within the rumen, and *Methanobrevibacter* spp. were predominant in the protozoa-associated methanogen communities [35]. As tannins are known to decrease the density of protozoa [36], perhaps, the dominance of methanogens belonging to the order Methanoplasmatales may be explained by the presence of tannins in the diets.

The protozoal communities were mainly comprised of *Ent*. *longinucleatum*, *Eud*. *maggii* and *Ep*. *caudatum*, representing the B-type rumen ciliate community [37]. In agreement with the previous studies, *Epidinium* spp. and *Eudiplodinium* spp. were also found in the rumen of sika deer [38]. Meanwhile, the diversity of fungal communities in the rumen of sika deer were also consistent with the results of Kittelmann et al. [9], who found the *Piromyces* sp. accounted for 20%.

The interactive relationship also showed the response of rumen microbiota and metabolic phenotypes in the rumen of sika deer fed tannin rich plants. Similar to the findings by Kang et al. [39], the present study showed that *Prevotella* spp. and unidentified bacteria within the family Veillonellaceae appeared in the co-occurrence network, indicating a potential metabolic link between the two groups of bacteria in the rumen of sika deer fed oak leaves. In agreement with our findings, Carberry et al. [8] also observed the negative associations between *Prevotella* spp. and isobutyrate, and isovalerate. The concentrations of isobutyrate and isovalerate were positively correlated with unidentified bacteria belonging to the family Veillonellaceae, suggesting that they may be the important producer of branched VFAs when tannins were fed to sika deer. In contrast, Mao et al. [40] found that *Anaerovibrio* spp., *Desulfovibrio* spp., *Leucobacter* spp. and *Moryella* spp. were positively correlated with isobutyrate and isovalerate in the feces of dairy cows. This difference may be caused by the distinct bacterial communities between rumen and fecal pellets [41].

The study also found that *Ep*. *caudatum* was negatively correlated with butyrate, whereas *Eud*. *maggii* was positively correlated with butyrate. This was interesting, given that butyrate is an important end product of rumen ciliate metabolism [42]. Michałowski et al. [43] found that *Eud*. *maggii* promoted butyrate production rate or concentration compared to defaunated animals. These results indicated that the composition of protozoa at the species level in the rumen may affect the energy supply to the host, as butyrate contributed to approximately 70% of the daily metabolic energy of ruminants [44].

Interestingly, methanogens belonging to the order Methanoplasmatales were negatively related to *Treponema* spp. A recent study showed that these methylotrophic methanogens mainly used hydrogen to reduce methanol and methylamines to methane [45]. However, *Treponema* spp. can utilize hydrogen to reduce carbon dioxide to acetate [46]. These findings suggested that the concentration and partial pressure of hydrogen in the rumen of sika deer may be one of the key factors affecting methanogenesis. Similar to recent findings [9,47], the present study also found a negative correlation between *Mbr*. *gottschalkii*, *Mbr*. *thaueri*, and *Mbr*. *millerae* with *Mbr*. *ruminantium*, indicating that the activity of different methanogens could play more important roles in methane production, rather than the density of methanogens [48]. Moreover, this may be due to the fact that these methanogens presumably compete for hydrogen as substrate [49,50]. The genome analysis also partially supported this notion. *Methanobrevibacter smithii* PS encodes a methyl coenzyme reductase II (*mcrII*), an isoenzyme of the methyl CoM reductase I (*mcrI*) enzyme, which was differentially regulated during growth to mediate methane formation at high partial pressure of hydrogen, while *Methanobrevibacter ruminantium* M1 contains only the *mcrI* system for the final methyl-CoM reduction step in methanogenesis [51,52]. In addition, *Piromyces* sp. A-BRL-3 and *Piromyces* sp. I-GRL-10 displayed positive and negative relationships with *Mbr*. *gottschalkii*, respectively. This may be related to the considerable variation in the fibrolytic ability the genus *Piromyces*, and in turn, resulted in the different substrates for methanogenesis [53]. Overall, the interactive analysis provided us novel insights into elucidating the symbiotic relationships between rumen microbiota and metabolism.

### Conclusions

In conclusion, this study demonstrated that the microbiota and fermentation in the rumen of sika deer fed tannin rich plants were altered. Different groups of methanogens were interactive with distinct rumen microbiota and fermentation products, suggesting the need to consider the different methanogen communities when developing strategies for mitigating methane emissions in ruminants. Such results would help us to understand the underlying mechanisms of decreased methane emission for ruminants by tannins, and improve strategies aiming to use tanniferous plants to reduce enteric methane emissions.
